# Study on the mechanical properties of granite responses of cyclic heating and water cooling considering microcosmic and energy

**DOI:** 10.1371/journal.pone.0312460

**Published:** 2024-11-01

**Authors:** Xiaokang Liang, Hanxiang Liu, Yong Yuan, Dong Zhu, Xiaowei Gu

**Affiliations:** 1 School of Mines, China University of Mining and Technology, Xuzhou, Jiangsu Province, China; 2 School of Transportation Engineering, Jiangsu Vocational Institute of Architectural Technology, Xuzhou, Jiangsu Province, China; 3 State Key Laboratory for Geomechanics and Deep Underground Engineering, China University of Mining and Technology, Xuzhou, Jiangsu Province, China; Central Mining Research Institute: Central Institute of Mining and Fuel Research CSIR, INDIA

## Abstract

Investigating the coupling effects of temperature levels and heating-water cooling cycles on the physical and mechanical responses of HDR (hot dry rock) is a vital issue during the exploitation of geothermal energy. In this study, the physical properties of granite specimens were measured first after each heating and water-cooling cycle. Then, uniaxial compressive tests were conducted on those granites to obtain their mechanical properties. With the increase in heating temperature (*T*) and cycles of heating and water cooling (*N*), P-wave velocity, uniaxial compression strength (UCS), and elastic modulus (*E*) showed a decreasing tendency, and the decrease of those four properties corresponding to T variation is greater than N variation. Due to the *α*-*β* phase transition of quartz happening at 573°C, the density UCS and E of granite decreased rapidly when the heating temperature increased from 450°C to 600°C at *N* = 1. With the increase of *T* and *N*, the failure mode of granite gradually changes from tensile failure to shear failure and, finally, comminute failure. The failure mechanism of granite gradually transfers from brittleness-dominated to ductility-dominated due to accumulated thermal damage. Finally, X-ray diffraction (XRD) and scanning electron microscope (SEM) were used to determine the damage mechanism of cyclic heating-cooling. The micro test results show that the high-temperature treatment changes the mineral composition and the microcracks number of the granite and finally affects the macroscopic physical and mechanical properties. The study conclusions of this manuscript are important for exploiting geothermal resources.

## Introduction

As the deterioration of the living environment is caused by the excessive consumption of fossil energy such as coal and oil, the development and utilization of clean and efficient green new energy have been a burning question worldwide to reduce carbon emissions [1–3]. Geothermal resources have been widely considered to be the primary new "green energy" for social development in the future due to their characteristics of sustainable development [4, 5]. In the geothermal mining process from hot dry rock, the rock undergoes repeated heating and water cooling [6, 7], which leads to the variation of physical and mechanical properties [8–10]. Besides, repeated heating and water cooling will change the crack distribution in the rock mass, finally influencing the permeability [11, 12]. Of all the lithology, granite is the most commonly studied because granite is the primary rock where geothermal resources exist [13–16]. Existing reports have indicated that the earth’s temperature grows by 25–50°C per kilometer in-depth, and some regions are characterized by a more significant geothermal gradient attaining 200°C/km due to specific geological settings [17, 18]. Thus, a wide temperature range of underground HDR, such as 150–650°C [19], has been accepted by some scholars [9, 20].

Studies show that temperature dramatically influences the physical and mechanical properties of granite. After one thermal treatment, the mass and density of granite experience a slight and then rapid decrease successively with the temperature increase with a temperature threshold of 400°C-600°C [21, 22]. When subject to cyclic heating and cooling, the mass and density loss rate of granite increased with a trend of “rapidly decrease, slowly decrease and finally nearly unchanged” with the increasing number of cycles [23]. The P-wave velocity decreases almost linearly with the temperature increase in the same cycle. However, the variation trend presents rapid decline and remains almost unchanged, with the number of cycles increasing at a constant temperature [23–26].

Due to the micro-crack induced by the thermal expansion of minerals [27], the uniaxial compression strength, tensile strength and elastic modulus of granite decrease with the temperature increase [27–29]. When subject to heating and cooling cycles, the mechanical parameters are strong relative to temperature and number of cycles [30]. Numerous pieces of literature [31–33] show the existence of a threshold of temperature and cycle times of uniaxial compression strength and elastic modulus. To explain the thermal deterioration mechanism, scanning electron microscope (SEM) [34–36], X-ray diffraction (XRD) [8, 9] and computerized tomography (CT) [27, 37] are the primary methods used to reveal the deterioration mechanism from the microcosmic viewpoint. Additionally, some damage variables calculated from variations of mechanical parameters [38] were put forward to describe the influence of temperature and cycles quantitatively.

However, few studies focus on the relationship between thermal damage and brittleness/ductility transition caused by heating and water-cooling cycles. Therefore, in this paper, uniaxial compression was conducted on granite exposed to high temperatures (150°C, 300°C, 450°C, 600°C, and 750°C) with various heating and water cooling cycles (1, 5, 10, 15) to obtain the mechanical properties. Then, the variation of thermal damage and elastic strain energy of the pre-peak stage were calculated. Finally, X-ray diffraction (XRD) and scanning electron microscope (SEM) were used to determine the damage mechanism of thermal treatment on macro mechanical properties from a micro perspective.

## Experimental setup

### Preparation of granite specimens

All the granite tested in this paper is derived from the same sizeable intact rock in Jiangsu Province, China. According to the recommendation of ISRM [39], the granite materials were processed into cylinder specimens in Φ50×100 mm dimension (flatness of end surface controlled within ± 0.02 mm) for a uniaxial compression test. Before the thermal treatment, the specimens were baked in a drying oven at 50°C for 24 hours, and then the basic physical and mechanical properties were tested. Three granites were left without subsequent thermal treatment as control groups. The average density and P-wave velocity of granite without thermal treatment is 2.65 g/cm^3^ and 4.47 km/s, and the uniaxial compression strength (UCS) and elastic modulus (*E*) are 82.4 MPa and 11.50 GPa.

**Fig 1:**
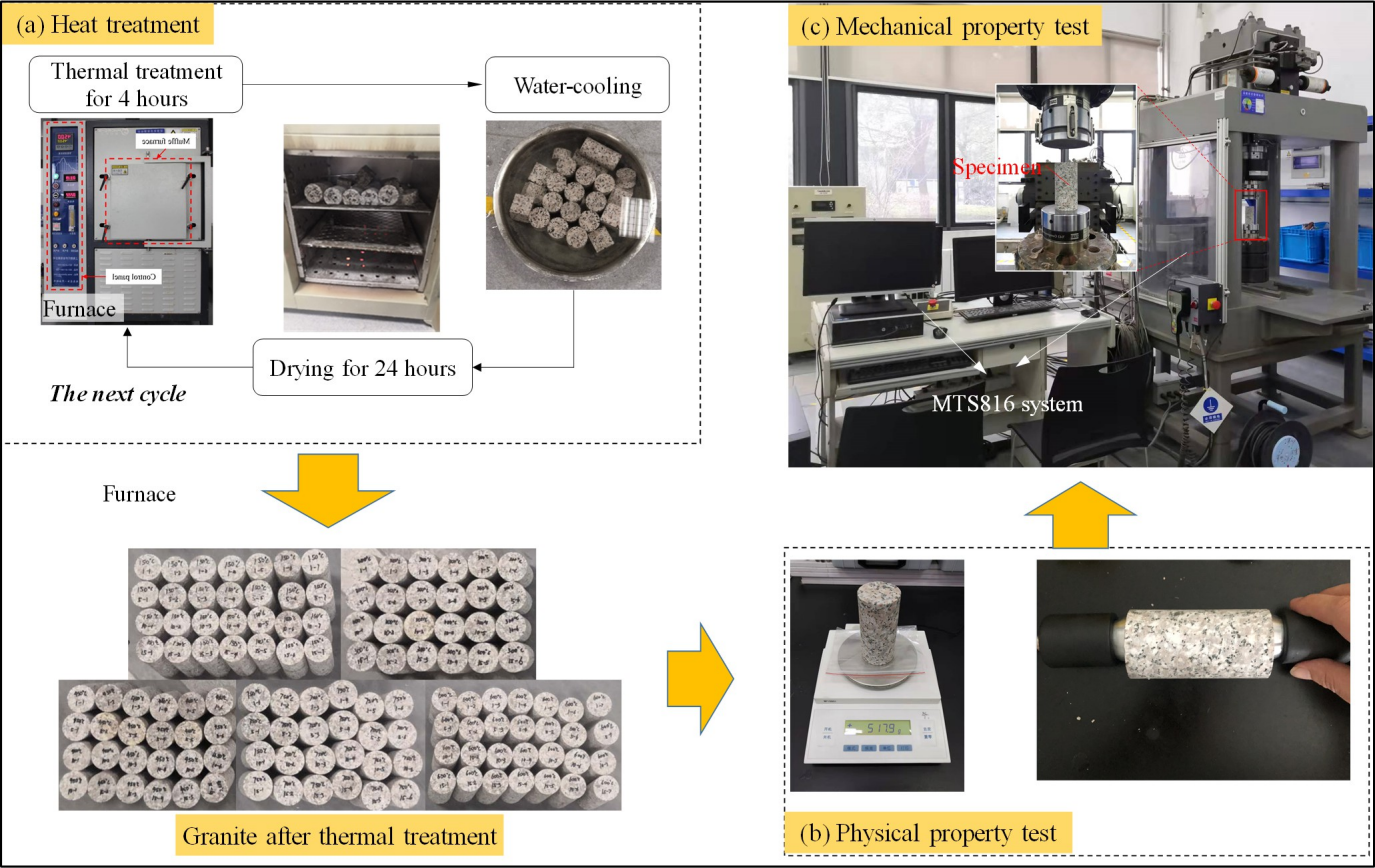
Step diagram of the cyclic heating-cooling treatment process of granite and experimental equipment used in this study.

After measuring the physical properties, the specimen experienced multiple heating and water cooling cycles following the steps shown in Fig 1. Firstly, the granite specimens were heated using a furnace (MXQ1700) with a rising rate of 10°C/min until the set treatment temperature (*T*). Then, the granite specimens would be kept at *T* for four hours in the furnace to ensure uniform heat treatment. After thermal treatment, the specimens were rapidly removed from the furnace and put in a container filled with running cold water for two hours to cool rapidly. After that, all granites were dried in a baker at 50°C for 24 hours and began the next heating-cooling cycle. The cycle is 1, 5, 10, and 15 for each temperature level.

### Test procedures

After heating and water cooling cycles, physical parameters were measured first; then, the uniaxial compressive test was implemented using a servo-hydraulic testing machine (as shown in Fig 1) to determine the influence on the mechanical properties of temperature and cycles. The axial stress was loaded by displacement control mode at 0.2 mm/min during the loading process. Finally, SEM and XRD were used to determine the microstructure and mineral composition of granite, which can effectively reveal the variation of the mechanical properties of rock from a micro perspective.

## Experiment results

As too many experimental conditions are involved in this paper, only the average value of experimental results under each condition is given. The average value of granite’s physical and mechanical properties after thermal treatment is listed in Table 1.

**Table 1:** Several physical and mechanical properties of granite before and after thermal treatment.

*T* (°C)	*N*	Before thermal treatment			After thermal treatment					
		*m* (g)	*V* (cm^3^)	*ρ* (g/cm^3^)	*m* (g)	*V* (cm^3^)	*ρ* (g/cm^3^)	*v*_p_ (km/s)	UCS (MPa)	*E* (GPa)
**Control groups**					524.83	198.05	2.650	4.470	82.40	11.50
**150**	1	524.40	197.39	2.651	521.85	197.82	2.638	4.397	79.39	10.89
	5	521.52	196.34	2.657	518.94	196.84	2.636	4.228	68.18	8.91
	10	522.24	196.59	2.656	519.54	197.22	2.634	4.194	61.38	7.42
	15	523.64	197.13	2.657	520.88	197.80	2.633	4.172	41.32	6.24
**300**	1	522.00	196.50	2.656	519.42	197.30	2.633	3.609	69.29	9.93
	5	523.27	197.00	2.657	520.62	197.93	2.630	3.165	61.39	8.56
	10	521.70	196.39	2.656	518.84	197.53	2.627	2.955	53.49	6.88
	15	523.61	197.10	2.656	520.60	198.57	2.622	2.925	39.31	5.08
**450**	1	521.64	196.36	2.657	518.35	199.29	2.601	2.612	62.22	8.4
	5	522.80	196.78	2.657	519.26	199.69	2.600	2.199	54.4	7.2
	10	522.46	196.69	2.657	518.78	200.17	2.592	2.080	43.48	5.45
	15	522.17	196.57	2.656	518.01	200.65	2.582	1.975	38.15	4.3
**600**	1	521.58	195.95	2.656	515.42	207.09	2.489	1.722	38.74	4.16
	5	523.73	197.16	2.662	516.07	209.09	2.468	1.185	32.48	3.93
	10	522.64	196.72	2.656	514.20	208.72	2.464	0.958	31.76	3.71
	15	523.55	196.97	2.657	514.83	209.25	2.460	0.742	30.04	3.31
**750**	1	523.92	197.52	2.658	516.41	210.15	2.457	1.292	25.75	2.43
	5	523.31	197.25	2.652	514.30	209.90	2.450	0.915	15.76	1.34
	10	521.63	196.30	2.653	510.95	210.24	2.430	0.713	11.47	0.84
	15	524.20	197.31	2.657	513.38	211.58	2.426	0.594	10.31	0.82

*m* is the specimen mass; *V* is the specimen volume; *ρ* is the specimen density; *v*_*p*_ is P-wave velocity; UCS and *E* are the uniaxial compression strength and elastic module of the specimen.

### Variation of density and P-wave velocity

**Fig 2:**
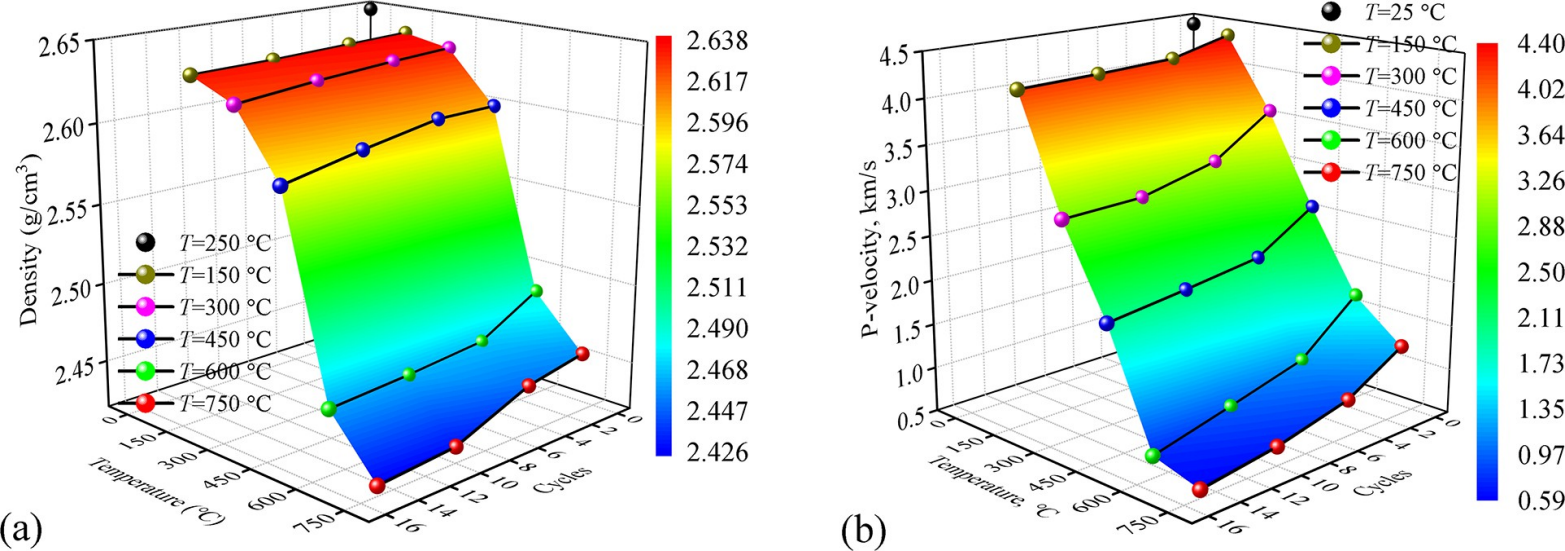
The variation of granite density and P-wave velocity in different temperatures and cycles. (a) Variation of density; (b) Variation of P-wave velocity.

The variation of density and P-wave velocity of granite are shown in Fig 2, and it is evident that both *T* and *N* influence physical properties. With the increases in *T*, granite density decreases first slowly then rapidly when *T* increases from 450°C to 600°C and finally slowly again when *T* ≥ 600°C. But the influence of *N* on density is less than *T*, as can be seen in Fig 2(A): when *T* = 150°C and 450°C, the density kept almost the same with *N* increase from 1 to 15; when *T* ≥ 600°C, the effect of *N* emerges when *N* ≥ 5, and the density decrease with the increase of *N*. Unlike the variation of density, P-wave velocity decreases almost linearly with the increase of *T* and *N*. To describe the influence on density and P-wave velocity of granite of cyclic thermal treatment, *T* and *N* were defined as two independent variables, with density and P-wave velocity as the dependent variables. By comparing the fitting degree of different equations, it is finally determined that bivariate quadratic equations were used. The fitting function of the fitting surface is listed in Table 2.

**Table 2:** The fitting function of density and P-wave velocity corresponding to with respect to *T* and *N*.

Function	$Y=z_{0}+a\cdot T+b\cdot N+c\cdot T^{2}+d\cdot N^{2}+f\cdot T\cdot N$						
Parameters	*z*_0_	*a*	*b*	*c*	*d*	*f*	*R* ^2^
**Density**	2.651	4.05e-5	-4.35e-4	-4.2e-7	3.92e-5	-3.46e-6	0.905
**P-wave velocity**	5.987	-0.0971	-0.0791	4.77e-6	0.0038	-5.74e-5	0.985

As demonstrated in the studies conducted by Yin et al. [40] and Gu et al. [41], with the increases in *T*, the bound water and structural water within granite gradually dissipate, leading to a weight reduction in granite. The disruption of mineral chemical bonds and the influence of thermal stress induce microcracks within granite, which not only enlarge its volume but also decrease its P-wave velocity. The combined effect of mass reduction and volume expansion results in a decrease in density. The drastic changes observed beyond 450°C are attributed to the volumetric expansion caused by the crystalline phase transition of quartz occurring above 573°C, further exacerbating volume enlargement and crack propagation. Furthermore, the sharp thermal expansion and cold shrinkage caused by water cooling will weaken the adhesive property among mineral particles, which may be the reason for the influence of *N*.

### Variation of mechanical properties

**Fig 3:**
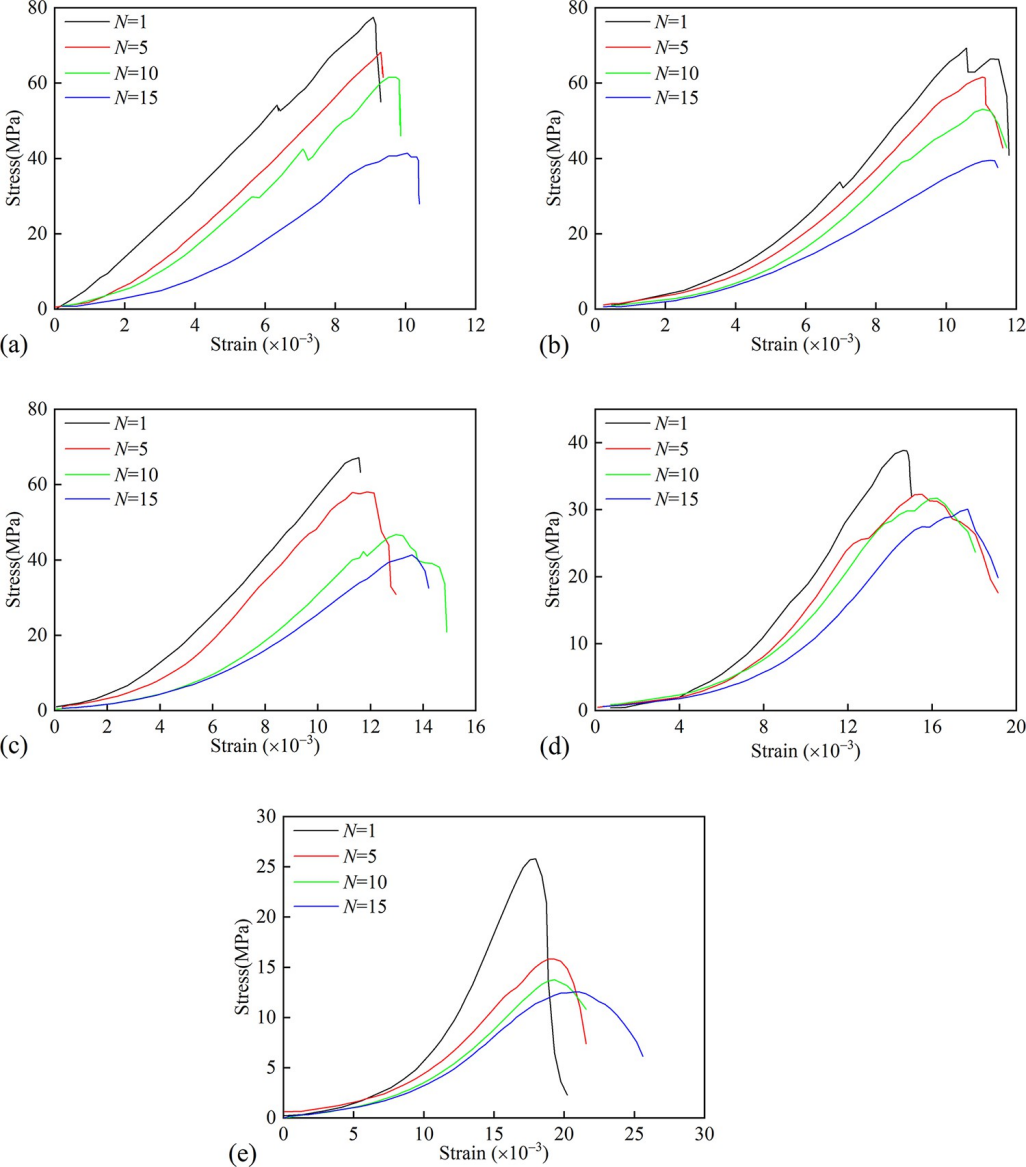
The stress-strain curve of granite after cyclic thermal treatment. (a)*T* = 150°C; (b) *T* = 300°C; (c) *T* = 450°C; (d) *T* = 600°C; (e) *T* = 750°C.

Fig 3 shows the stress-strain curve of granite under uniaxial compressive after multiple heating and water cooling cycles. As shown in Fig 3, the stress-strain curve can be divided into four stages: crack compaction stage (stage 1), elastic deformation stage (stage 2), yield stage (stage 3) and soften stage (stage 4). For *T* = 150°C, 300°C and 450°C, stages 1, 3 and 4 increase significantly with the increase of the *N*, but oppositely for stage 2; for *T* = 600°C and 750°C, all those variations are apparent when *N≤*5. The above analysis means that cyclic heating and cooling enhance the ductility of granite, and the effect of *N* is weakened at higher *T*.

**Fig 4:**
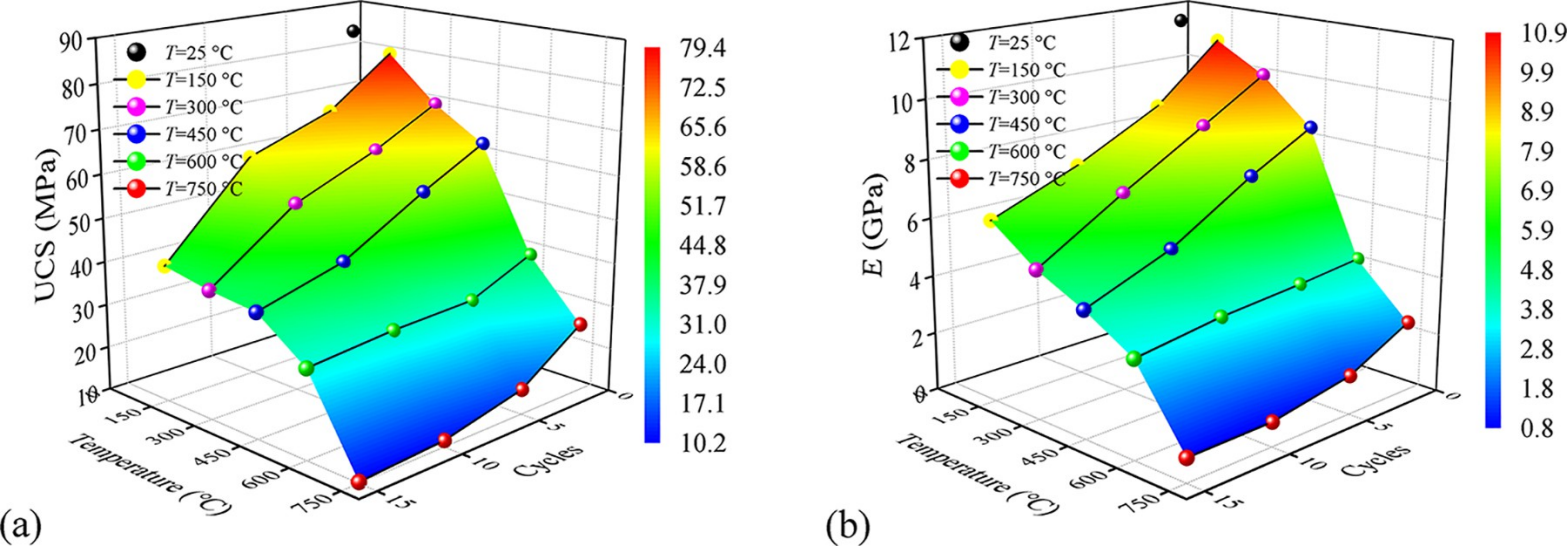
Variation of UCS and *E* of granite after thermal treatment with different *T* and *N*. (a) the coupling effect of T and N on the variation of UCS; (b) the coupling effect of T and N on the *E* variation.

Fig 4 shows the variation of UCS and *E* with the coupling increase of *T* and *N*. As shown in Fig 4(A), the UCS decreases with the increase in *T* and *N* but has tiny differences at different temperatures. When *T* is 150°C, 300°C and 450°C, the UCS of granite drops dramatically with the increase of cycles. For instance, the UCS at 150°C are 79.4 MPa, 68.2 MPa, 61.4 MPa and 41.3 MPa, respectively, corresponding to *N* = 1, 5, 10, and 15. When *T* >450°C, the UCS of granite still drops when *N* increases from 1 to 5, and then almost unchanged when *N* increases from 5 to 15. That means a threshold of *N* exists: when *N* is greater than the threshold, the influence of *N* on UCS can be ignored, which is consistent with Li’s study [42]. Similar to the influence of *T*, for *N* = 1, 5 and 10, the UCS of granite decreases obviously with the increase of *T*. But for *N* = 15, the UCS of granite decreases tiny (from 41.32 MPa to 30.02 MPa) with *T* increases from 150°C to 600°C and then sharply (from 30.02 MPa to 10.31 MPa) when *T* increased from 600°C to 750°C.

The variation of *E* is similar to UCS as shown in Fig 4(B): with the increasing *T* and *N*, the *E* decreases from 11.89 GPa to 0.82 GPa from *T* = 150°C, *N* = 1 to *T* = 750°C, *N* = 15. In addition, the degradation effect of *T* after one cycle is more significant than after more cycles: when *N* = 1, the difference value of *E* is 9.55 GPa, and that value is 7.57 GPa, 6.58 GPa and 5.42 GPa at *N* = 5, 10 and 15. To describe the influence of UCS and *E* of granite of cyclic thermal treatment, with *T* and *N* as two independent variables, UCS and *E* as the dependent variables, then the fitting functions and were obtained as shown in the following list in Table 3:

**Table 3:** The fitting function of UCS and *E* corresponding to with respect to *T* and *N*.

Function	$Y=z_{0}+a\cdot T+b\cdot N+c\cdot T^{2}+d\cdot N^{2}+f\cdot T\cdot N$						
Parameters	*z*_0_	*a*	*b*	*c*	*d*	*f*	*R* ^2^
**Density**	86.2905	-0.0112	-3.2705	-9.994e-5	0.0179	0.0031	0.979
**P-wave velocity**	12.1785	-0.0024	-0.5350	-1.467e-5	0.0057	-4.777e-4	0.976

### Variation of elastic strain energy

**Fig 5:**
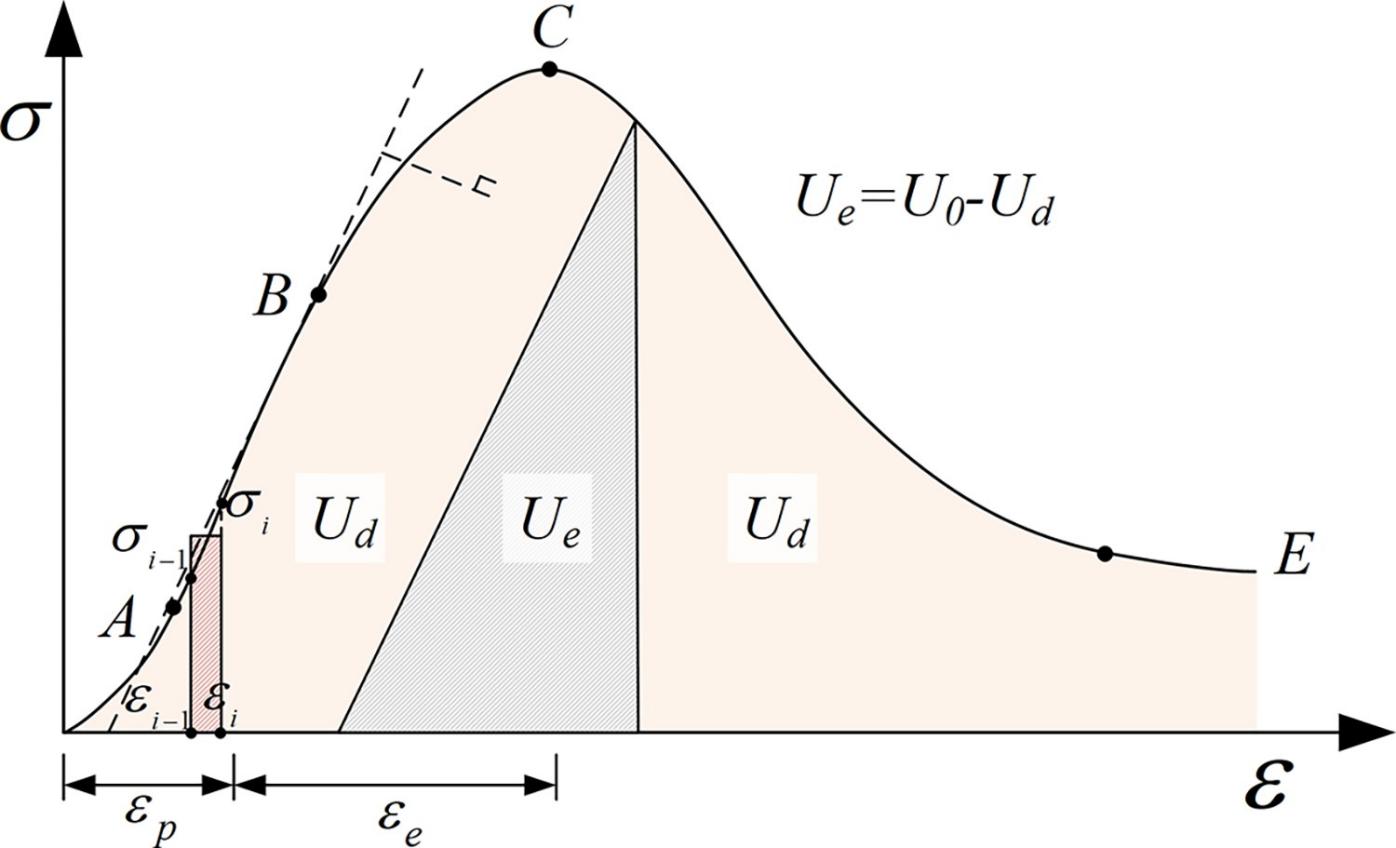
Relationship of elastic strain energy and dissipation energy per unit volume. *U*_*d*_ is the dissipative energy and *U*_*e*_ is the elastic energy.

**Fig 6:**
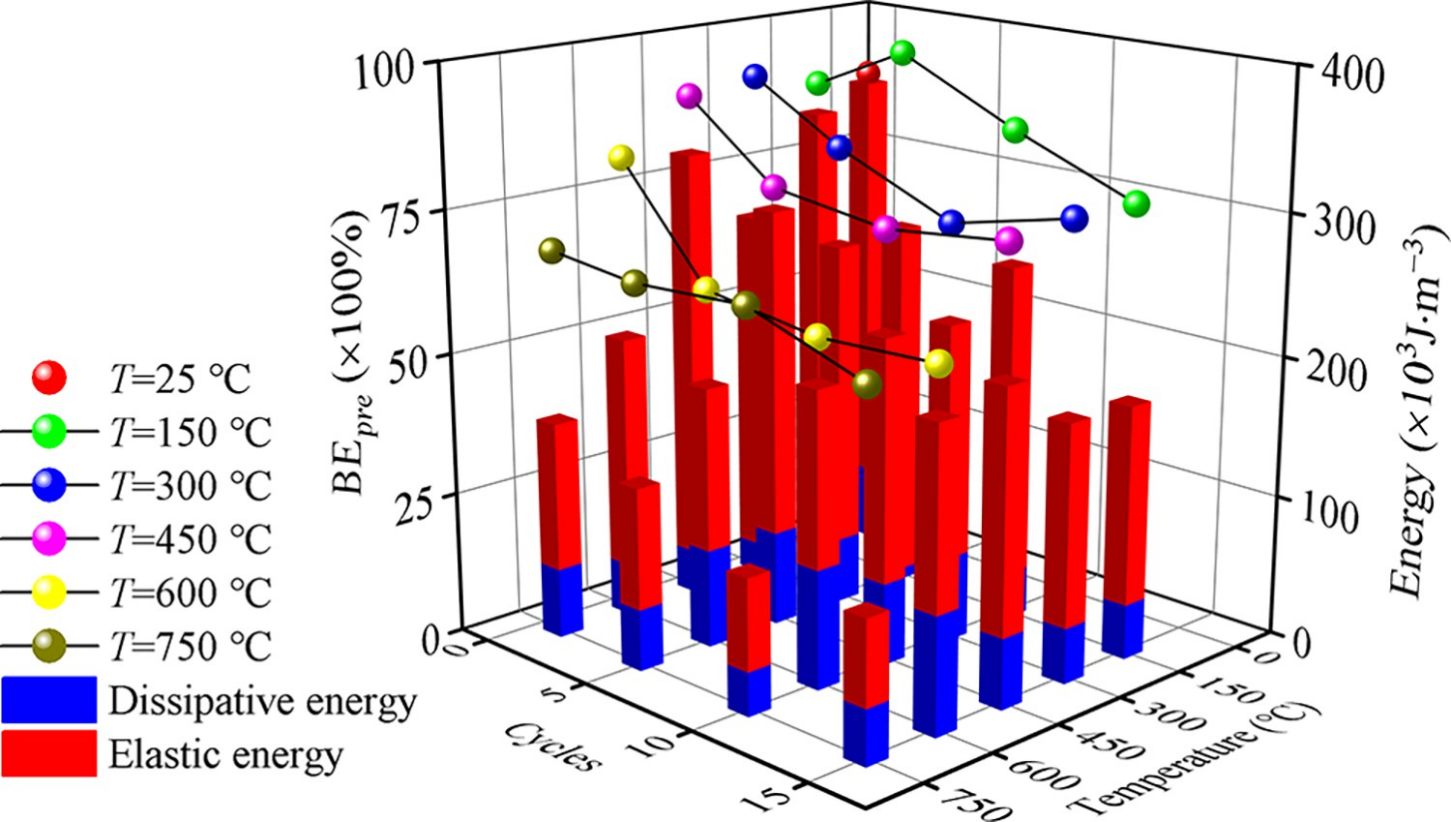
The variation of energy evolution in the pre-peak stage of granite after thermal treatment under uniaxial compression. *BE*_*pre*_ is the ratio of elastic energy to dissipative energy.

The above analysis shows that thermal treatment leads to the plasticity enhancement and elastic reduction of granite and finally leads to the transformation of failure mode. To quantitatively describe the brittle-ductility transfer characteristics, the strain energy in the pre-peak stage, as shown in Fig 5, of granite with different *T* and *N* is calculated following Meng’s method [43]. The result is shown in Fig 6. As shown in Fig 6, with the increase of *T* and *N*, both the input energy and *BE*_*pre*_ decrease. For granite without thermal treatment, *BE*_*pre*_ equals 0.89, which means the lithology of granite at 25°C tends to be elastic. For *T* = 750°C, the *BE*_*pre*_ is 0.68, 0.66, 0.67 and 0.60 corresponding to *N* = 1, 5, 10 and 15, which can explain granite’s failure mode at *T* = 750°C.

### Variation of failure mode

**Fig 7:**
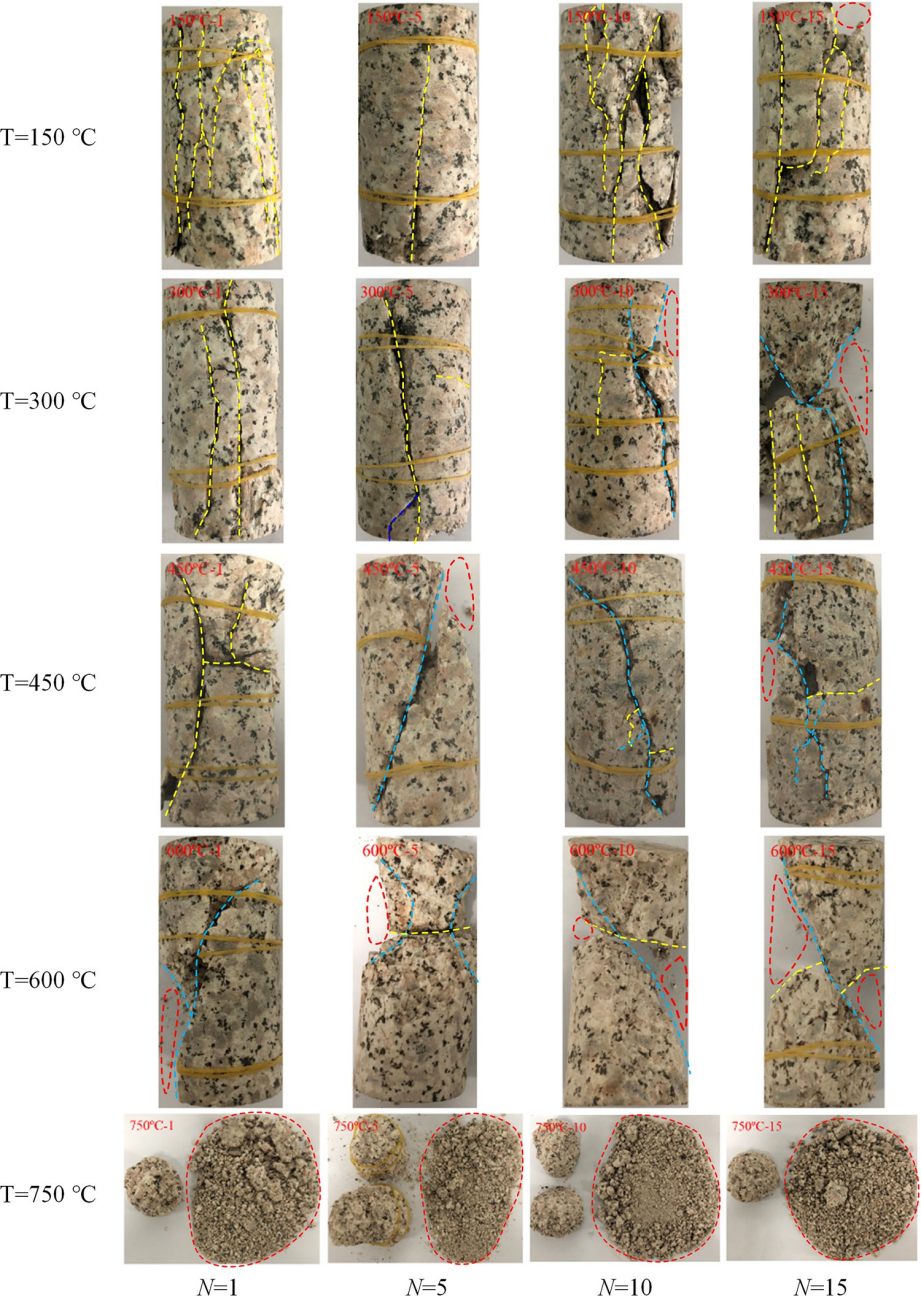
Ultimate failure mode of granite with thermal treatment after uniaxial compression.

The final failure mode of granite specimens after thermal treatment with different *T* and *N* are listed in Fig 7, in which the yellow and blue dots are used to depict the tensile and shear cracks, respectively, and the red ring is used to depict peeling pieces. As shown in Fig 7, when *T* = 150°C, the dominant crack in granite is a tensile crack along the axial loading direction at *N* = 1. 5, 10 and 15 and in the top-right part of the specimen peels fragment. When *T* = 300°C and 450°C, the dominant crack remains tensile at *N* = 1, 5 and *N* = 1, respectively. Furthermore, when *N* = 10 and 15, fragments peel off in the top-right part. When *T* = 600°C, the dominant failure cracks transform into shear cracks even at *N* = 1, and large fragments peel off specimens. When *T* = 750°C, the failure of the specimen still results from shear cracks, but because of the enhancement of plastic, the failed specimen can no longer remain intact and become completely broken. The failure mode changes from brittle tensile failure mode to ductile shear failure mode gradually with the increase in *T*. With the increase of *N*, the shear failure happens at 600°C, 450°C, 300°C and 150°C, corresponding to *N* = 1, 5, 10 and 15.

According to the contents in sections 4.2 and 4.3 below, under the action of high temperature, the dehydration, phase transformation, chemical bond breaking and thermal stress of the mineral composition inside the granite lead to changes in the pore structure and ultimately affect its physical and mechanical properties. With the increase of temperature and cycle times, the number of cracks caused by thermal damage increases and the fracture network is formed, which leads to the enhancement of the ductility of granite, and the shear failure is easier to occur. It is further indicated that the rocks gradually change from brittleness to ductility under the action of cyclic high-temperature water cooling. The increase of ductility makes the granite withstand greater axial deformation, while the expansion and increase of microcracks destroy the integrity of the granite, thus significantly reducing the strength of the rock.

### Variation of thermal damage

**Fig 8:**
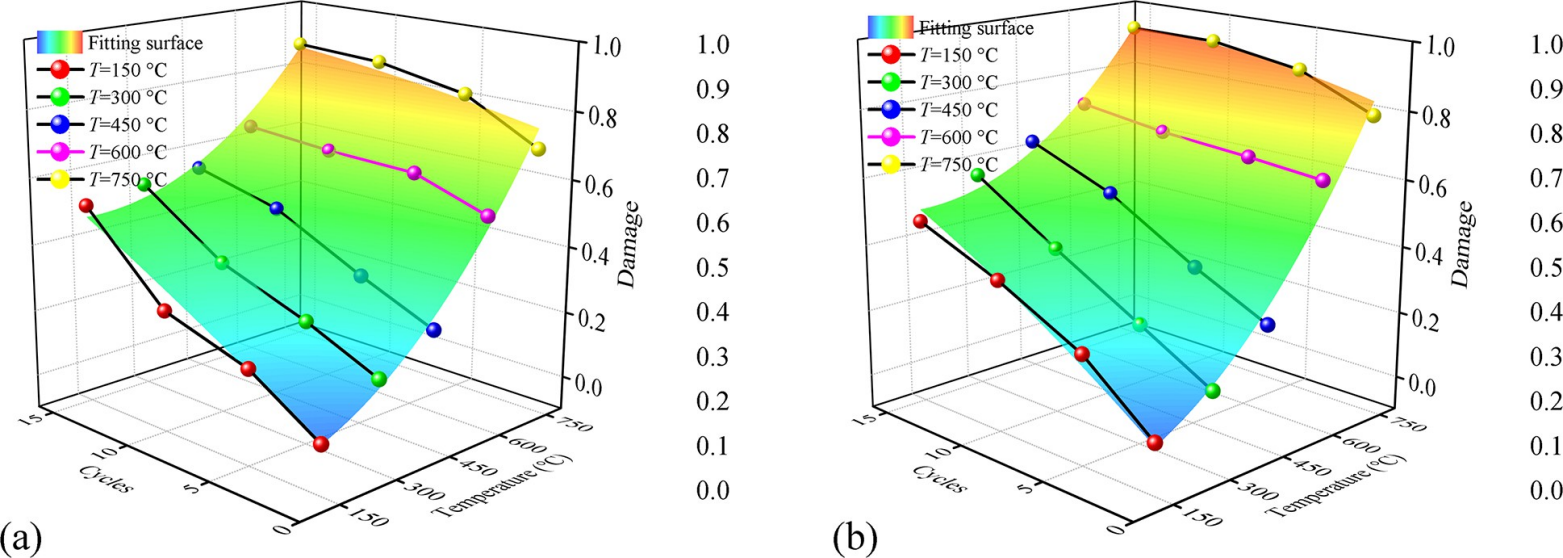
The variation of damage calculated by UCS and *E*, respectively. (a) The variation of *D*_UCS_ and (b) *D*_*E*_.

The variation of UCS, *E* and P-wave velocity of rock can be used to calculate the damage factor of rock caused by thermal treatment [44], as list in Eqs (1) and (2).

$$D_{\mathrm{UCS}}=1-\frac{{\mathrm{UCS}}_{T∼N}}{{\mathrm{UCS}}_{0}}$$ (1)

$$D_{E}=1-\frac{E_{T∼N}}{E_{0}}$$ (2)

where *D*_UCS_ and *D*_*E*_ are the damage estimated by UCS and *E*; UCS_*T*~*N*_ and *E*_*T*~*N*_ are the UCS and *E* of granite after thermal at unique *T* and *N*; UCS_0_ and *E*_0_ are the UCS and *E* of granite at *T* = 25°C and *N* = 1. The values of *D*_UCS_ and *D*_*E*_ calculated by Eq (1) and (2) are plotted in Fig 8, and the equations of the fitting surface are listed in Table 4

**Table 4:** The fitting function of *D*_UCS_ and *D*_*E*_ corresponding to *T* and *N*.

Function	$Y=z_{0}+a\cdot T+b\cdot N+c\cdot T^{2}+d\cdot N^{2}+f\cdot T\cdot N$						
Parameters	*z*_0_	*a*	*b*	*c*	*d*	*f*	*R* ^2^
***D***_**UCS**_	-0.0347	1.3405e-4	0.0392	1.197e-6	-2.142e-4	-3.665e-5	0.979
***D***_**E**_	-0.0599	2.091e-4	0.0466	1.276e-6	-4.990e-4	-4.158e-5	0.976

As shown in Fig 8(A) and 8(B), the fitting surface of *D*_UCS_ and *D*_*E*_ presents a concave surface, which is contrary to the fitting surface of UCS (Fig 4). As shown in Fig 8, the *D*_UCS_ and *D*_*E*_ increase with the increase in *T* and *N*. At the same *N*, both *D*_UCS_ and *D*_*E*_ increase gently and then sharply with the increase of *T*, and the difference value between each *T* decreases with the increase of *N*. With the same *T*, both and *D*_*E*_ increase almost linearly with different *N* and the difference value between each *N* decreases with the increase of *T*. The variation of *D*_UCS_ and *D*_*E*_ demonstrates greater *T* and *N* weak the degradation effect of each other.

**Fig 9:**
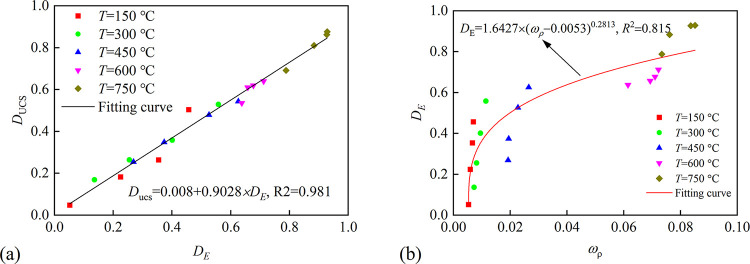
The relationship between damage. (a) The relation between *D*_UCS_ and *D*_*E*_; (b) The relation between *D*_*E*_ and *ω*_*ρ*_.

To verify the accuracy of each damage, the relationship between *D*_UCS_ and *D*_*E*_ is plotted in Fig 9(A); the function of fitting curve manifests the accuracy and rationality of applying *D*_UCS_ and *D*_*E*_ depicting damage resulting from thermal treatment. But sometimes, it is difficult to test the mechanical properties of rock, so it is necessary to determine the relationship between physical and mechanical properties. As shown Fig 9(B), *D*_*E*_ is strong relation to *ω*_*ρ*_ (we defined *ω*_*ρ*_ = density reduction/original density), and a power function can describe the relation well, as shown in Fig 9(B).

## Microscopic test results

### Analysis of mineralogy ingredient variation

**Fig 10:**
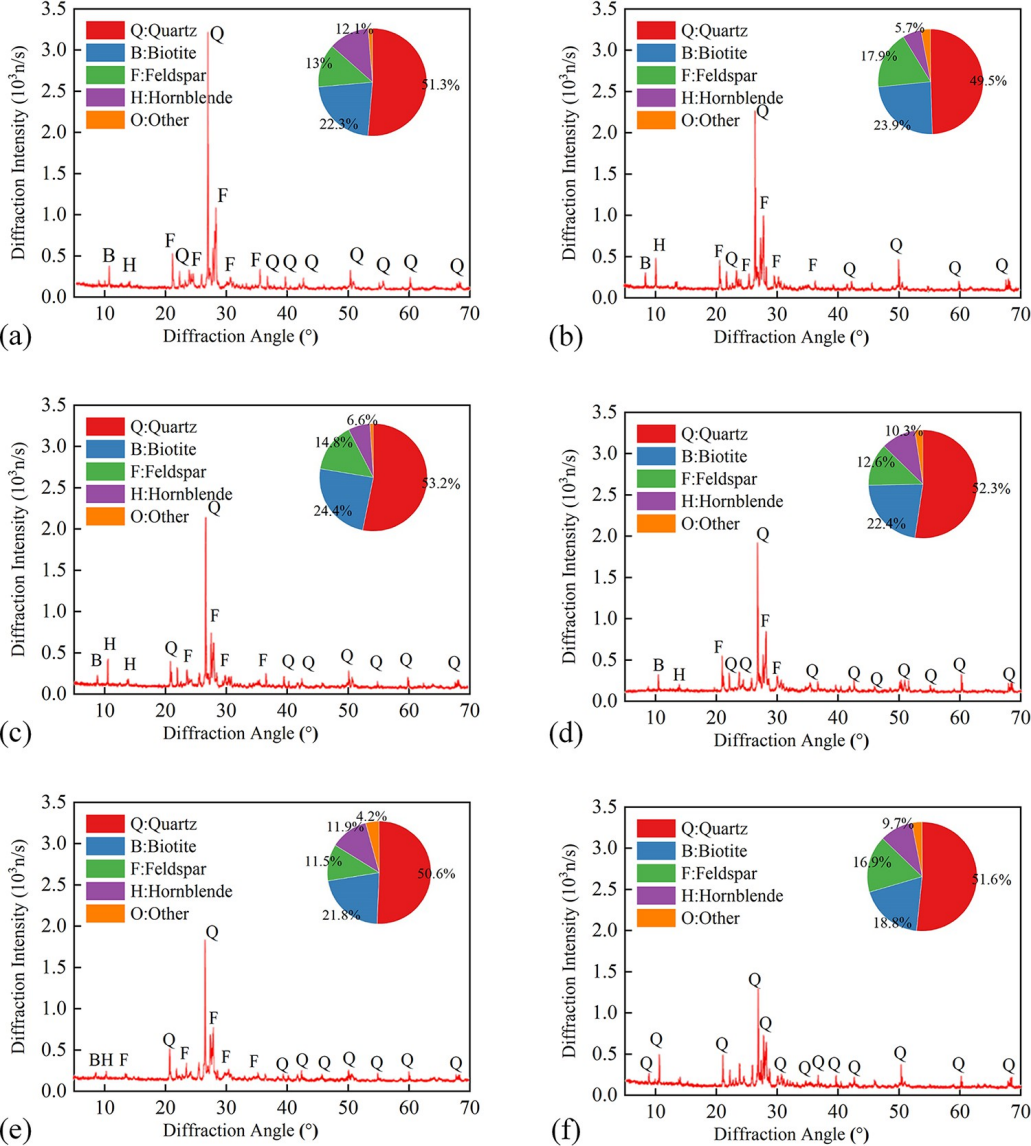
Diffraction patterns of granite specimen at different *T*. (a) *T* = 25°C; (b)*T* = 150°C; (c) *T* = 300°C; (d) *T* = 450°C; (e) *T* = 600°C; (f) *T* = 750°C.

Previous studies [45–49] have demonstrated that the difference in microscopic material composition leads to the difference in the macroscopic properties of rock. The diffraction patterns of granite specimens after one heating-cooling cycle at different *T* are shown in Fig 10. As evident from Fig 10, after heating with different *T*, the mineral composition of granite remains quartz, feldspar, biotite, and amphibole. However, with the increase in *T*, the content of primary minerals decreases, as observed in the pie charts. Besides, the diffraction intensity of quartz and feldspar gradually diminishes, which suggests that the high temperatures induce alterations of the minerals structure. When the temperature surpasses 573°C, triggering an *α*-*β* phase transformation of quartz. Additionally, the high-temperature treatment causes thermal expansion of mineral grains, leading to the formation of microcracks, which breaks the integrity and crystallinity of the mineral crystals, resulting in a reduction in diffraction intensity.

**Fig 11:**
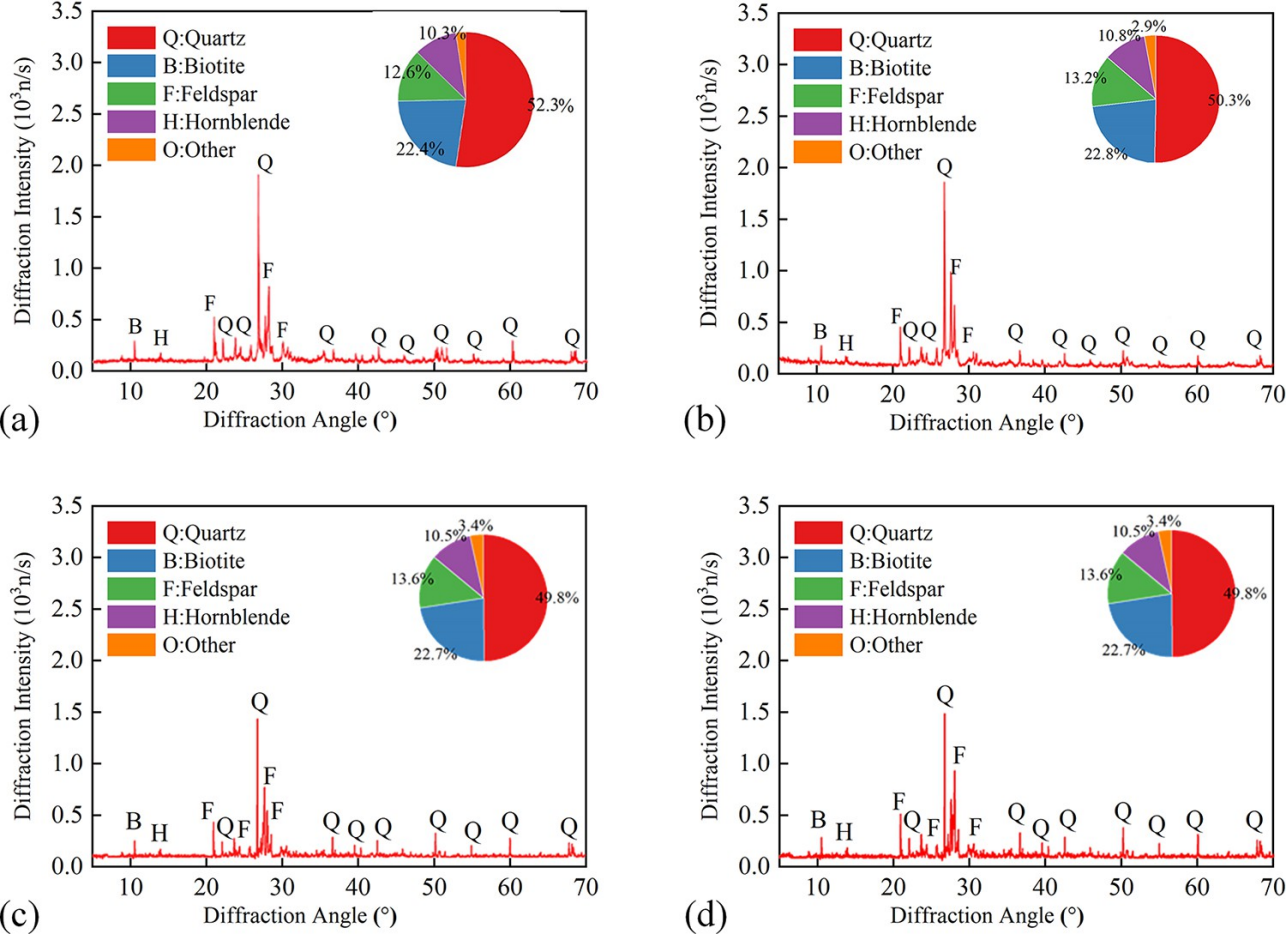
Diffraction patterns of granite specimen at 450°C after different heating-cooling cycles. (a) *N* = 1; (b) *N* = 5; (c) *N* = 10; (d) *N* = 15.

Fig 11 shows the diffraction patterns of granite at 450°C after different heating-cooling cycles. The mineral composition of granite remains almost unchanged with increasing *N*. However, a slight decrease in the diffraction intensity of granite and feldspar is observed. This phenomenon is primarily attributed to the fatigue damage of the minerals due to cyclic heating and water cooling. This process degrades the crystallinity of the minerals and ultimately results in a decrease in diffraction intensity.

### Analysis of SEM result

**Fig 12:**
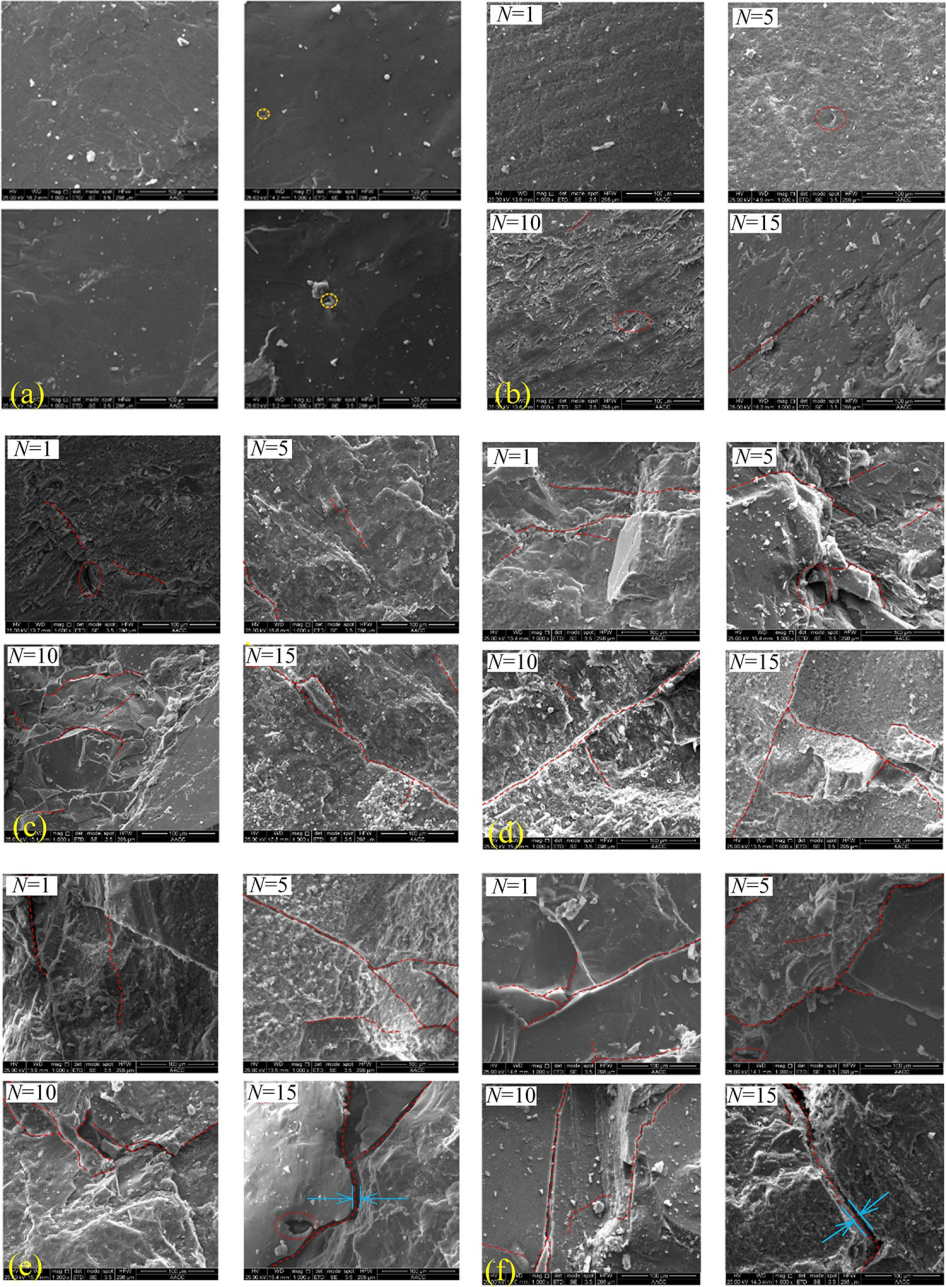
Evolution of microstructure in granite samples after thermal treatment. (a) *T* = 25°C; (b)*T* = 150°C; (c) *T* = 300°C; (d) *T* = 450°C; (e)*T* = 600°C; (f) *T* = 750°C.

Before the mechanical test, according to the number of groups of thermal treatment, the same number of small pieces with a size of about 5 mm was taken from the same granite block, and those small pieces were conducted with cycle high-temperature water-cooling treatment. After heat treatment and water cooling, the SEM tests were carried out. When selecting small granite pieces for the SEM test, the small pieces with similar morphology and color characteristics were selected to ensure the reliability of test results. The microstructure of granite specimens was observed by SEM with 1000 magnification and shown in Fig 12. The yellow and red dot lines depict holes and fissures, and blue arrows represent crack width.

As shown in Fig 12(A), only a small-scale spot and mineral clastic can be observed on the surface in vision, indicating that the natural granite is almost intact.

At 150°C, as shown in Fig 12(B), some holes can still be observed after 1~10 cycles, and only short micro-fissures can be observed in the lower-left part of the vision after 15 cycles. The microcosmic surface looked rougher than *T* = 25°C, and roughness increases with the number of cycles. In the 4th picture of Fig 12(B), some large mineral particles can be observed in the upper-right and lower-middle region of vision, and the microcosmic surface became uneven.

Fig 12(C) shows that when *T* = 300°C, micro-fissures increase with cycles. After one cycle, only two short cracks and one large hole are observed. After 5 and 10 cycles, the number of short cracks increases obviously, and some pits appear on the surface; especially in the 3rd picture of Fig 12(C), large particles can be seen around the fracture. When *N* = 15, a crack throughout vision from the upper left to the lower right part appeared, indicating that the increase of *N* will make the short fissure connect to penetrate the crack. However, in the picture of 15 cycles, no large particle is found, which may contribute to the large number of heating-cooling cycles leading to granular fragmentation.

As shown in Fig 12(D), the long crack is generated after a few cycles (1 and 5 cycles) at 450°C, and significant pits appear even after one cycle. With the increase of *N*, long cracks begin to penetrate the field of vision, and eventually, a network of cracks forms and destroys the integrity of the rock. Also, some particles can be observed around the fracture in the 2nd and 3rd pictures of Fig 12(D). Fig 12(C) and 12(D) show the generation and development of cracks from short to long in granite.

When *T* reaches 600°C and 750°C, a notable change of microcrack is that the opening of the crack increases after 10 and 15 cycles, as marked by the blue arrow, which may result from the phase transition of quartz at 573°C and the increasing cycles makes the phase transition irreversible. The generation and development of long cracks may be the reason for the dramatic volume variation. Fig 12(F) shows that large amounts of big mineral particles are shed, which can explain the significant mass variation when *T* ≥ 600°C.

When *T* is in the range of 150–450°C, the uneven expansion of minerals serves as the primary cause for the initiation and propagation of microcracks. However, due to the relatively low thermal stresses at these *T*, the resulting microcracks exhibit narrow opening widths. When *T* = 600°C and 750°C, mineral phase transformations and uneven thermal expansion whill generate significantly higher thermal stresses, leading to an increase in both the opening widths and the number of microcracks. Furthermore, the fatigue damage resulting from increased cycles further intensifies the propagation of these cracks.

## Conclusion

(1) Both the *T* and *N* influence density and P-wave velocity of granite, but the influence of *N* is less than *T*. *T* = 450°C is the threshold of temperature for density: when *T*≤ 450°C, the density of granite increases slowly with the increase of *T* and then a dramatic decrease happens when *T*>600°C. Unlike density, P-wave velocity decreased almost linearly with the increase of *T* and *N*. A bivariate quadratic function of *T* and *N* can well describe the variation of P-wave velocity.

(2) Thermal treatment has an excellent degradation effect on the mechanical properties of granite—the UCS and *E* decrease with the increased set temperature and number of cycles. To describe the degradation effect of cyclic heating and cooling, UCS and *E* were used to calculate the damage caused by thermal treatment. When *T* is 150°C, 300°C and 450°C, the UCS and *E* of granite dropped dramatically with the increase of cycles; When *T* >450°C, the UCS and E of granite still dropped dramatically for *N* increase from 1 to 5, and then almost unchanged with N increase from 5 to 15. Furthermore, with the increase of *T* and *N*, the characteristic of granite gradually changes from elastic to plastic as the decrease in *BE*_*pre*_.

(3) Both *T* and *N* greatly influence the failure mode of granite. When *T* = 50°C, the failure mode of granite is tensile failure mode; for *T* = 300°C and 450°C, the failure mode translates from tensile failure mode to shear failure mode at larger *N*; for *T* = 600°C, the failure mode of granite was still shear mode but with larger pieces flaking and finally splitting into halves. Crushing failure mode occurred when subjected to high temperature (T = 750°C).

(4) XRD and SEM results show *T* and *N* significantly influence mineral composition and microstructure. The increased *T* and *N* lead to a decrease in the diffraction intensity of quartz. However, cyclic heating and water cooling slightly influence mineral composition type and proportion. For *T*≤ 450°C, the increase of *T* and *N* leads to the rise in quantity and length of micro-fissures and makes the coalescence of short fissures. For *T* ≥600°C, penetration cracks occur even with *N* = 1 and the increase of *N* leads to fissures opening increase. The evolution process of micro-fissures observed by SEM can explain the variation of density and P-wave velocity. The coalescence of fissures destroys the integrity of the granite, ultimately resulting in the degradation of mechanical properties.
